# In a Budapesth Hospital

**Published:** 1921-01-15

**Authors:** 


### IN A BUDAPESTH HOSPITAL.

### BY A SPECIAL CORRESPONDENT.

To-day I visited the State Asylum in Budapesth—an institution started twenty years ago by the State to provide for all penniless children and foundlings. Every child whose parents cannot provide for it, or who has no parents, has a right to be admitted, and, if it be ill, it is given medical treatment. Mothers of infants, too, are admitted, so that they may suckle their children.

The institution, however, has only 362 beds, and since, even in peace time, there were many hundreds of destitute in Hungary, the plan was adopted to send any overflow of children to peasants in the country, and to pay the peasants for their trouble. That was quite a good scheme, and worked admirably before the war; but, since the war, the peasants have grown comparatively rich, and not only rich but selfish, and now they refuse to take the children, even though they know that children in Budapesth are dying of starvation and are receiving help from foreign countries. Now the "Asylum" is overcrowded, and often two or three children must sleep in one bed.

The institution is not only an asylum, but also a hospital, for many of the children admitted are ill and diseased, and there are hospital wards where treatment is carried out. There can be no doubt that the institution has done, and is still doing, excellent work, but, since the war, lack of funds has ever more and more handicapped its efforts. Dr. Torkay, the director, is a man of great energy and enthusiasm, but for the last few years he has been fighting a losing battle. With the funds available the children can be neither adequately fed nor clad. On the beds there are only paper sheets, and the pillow-cases are not half big enough to hold the pillows, and sheets and garments have been patched and repatched till they are all patch together. I went into the mending-room, and there I saw ragged garments such as I have never seen before. Many articles were quite curiosities in raggedness, and seemed quite beyond redemption, though several men and women were doing their best to repair them.

Not only the children and their mothers but also the maids and workers in the asylum are inadequately clad, and many of the nurses and servants had no stockings, and some neither stockings nor boots. One cannot help admiring the pluck, courage, and patience of those who are working for humanity in such very depressing and adverse circumstances. I have seen many needy institutions in Hungary, but the State Asylum is, I think, the neediest I have seen.

